# Low risk squamous cell carcinoma and appropriate follow up

**DOI:** 10.1002/ski2.364

**Published:** 2024-03-19

**Authors:** Dáire Goodman, Gary Fenn, Brian Pierce, Rhona Thuillier, Hannah Glavin, Roisin Dolan

**Affiliations:** ^1^ Department of Plastic and Reconstructive Surgery St Vincent's University Hospital Dublin Ireland; ^2^ Department of Histopathology St Vincent's University Hospital Dublin Ireland

## Abstract

A recent article in the BJD postulated that it may be "Time to reconsider skin cancer‐related follow‐up visits". In our unit, we too have been seeing too many patient's unnecessarily and we put in place measures to reduce the numbers of outpatient appointments thereby diverting the resources saved into professional development.

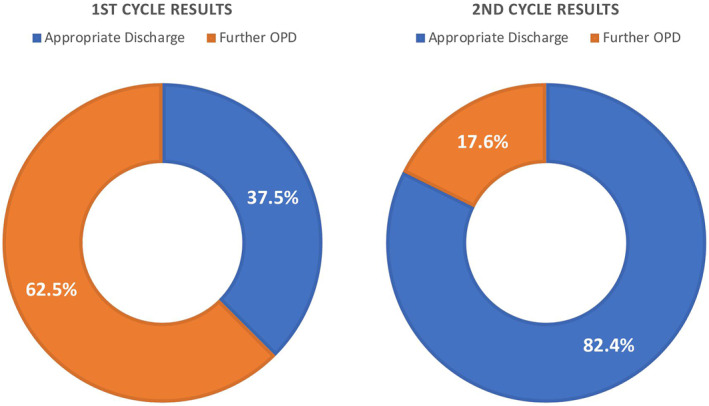

Dear Editor, We read with interest your recent article on skin cancer related follow‐up^1^ and questioned if we were surveilling keratinocyte carcinoma (KC) patients too frequently.

Nonmelanoma skin cancer is the most common malignancy affecting Caucasians and over the last 20 years its incidence has increased worldwide.^2^, ^3^ The most recent audit from the National Cancer Registry of Ireland showed that almost 11 000 invasive skin cancers were diagnosed between 2011 – 2015 with cutaneous squamous cell carcinoma (cSCC) comprising 27.7% of these cancers. A 2.8% average annual percentage increase of cSCC incidence for both male and female was observed between 2012 and 2015 and the incidence is projected to increase.^4^

With this increase in cSCC incidence, we are receiving ever more referrals to our Outpatients Department (OPD) regarding cSCC assessment and excision. We used the revised British Association of Dermatologist (BAD) guidelines^5^ as our audit standard for the management for cSCC. These guidelines define low‐risk cSCCs as a tumour ≤ to 20 mm in diameter, tumour thickness ≤4 mm, tumour invasion into dermis only, no lymphovascular or perineural invasion identified in the specimen and only of a well‐differentiated or moderately‐differentiated histology. Low‐risk cSCCs must have a clear pathology margin (≥1 mm) in all dimensions when excised, and the patient must beimmune‐competent. British Association of Dermatologist guidelines recommend that low‐risk cSCC need not be followed up after single post‐treatment appointment once completely excised. Our audit reviewed clinical practice and management of low‐risk cSCC patients in our unit, adherence to BAD guidelines and appropriate discharge following post‐op review.

During our first audit cycle, we identified 24 patients with low‐risk cSCCs from July and August 2022. 15 (62.5%) of these patients were inappropriately offered follow up appointments despite being adequately excised. 9 (37.5%) of these patients were discharged appropriately according to BAD guidelines (Figure 1).

**FIGURE 1:**
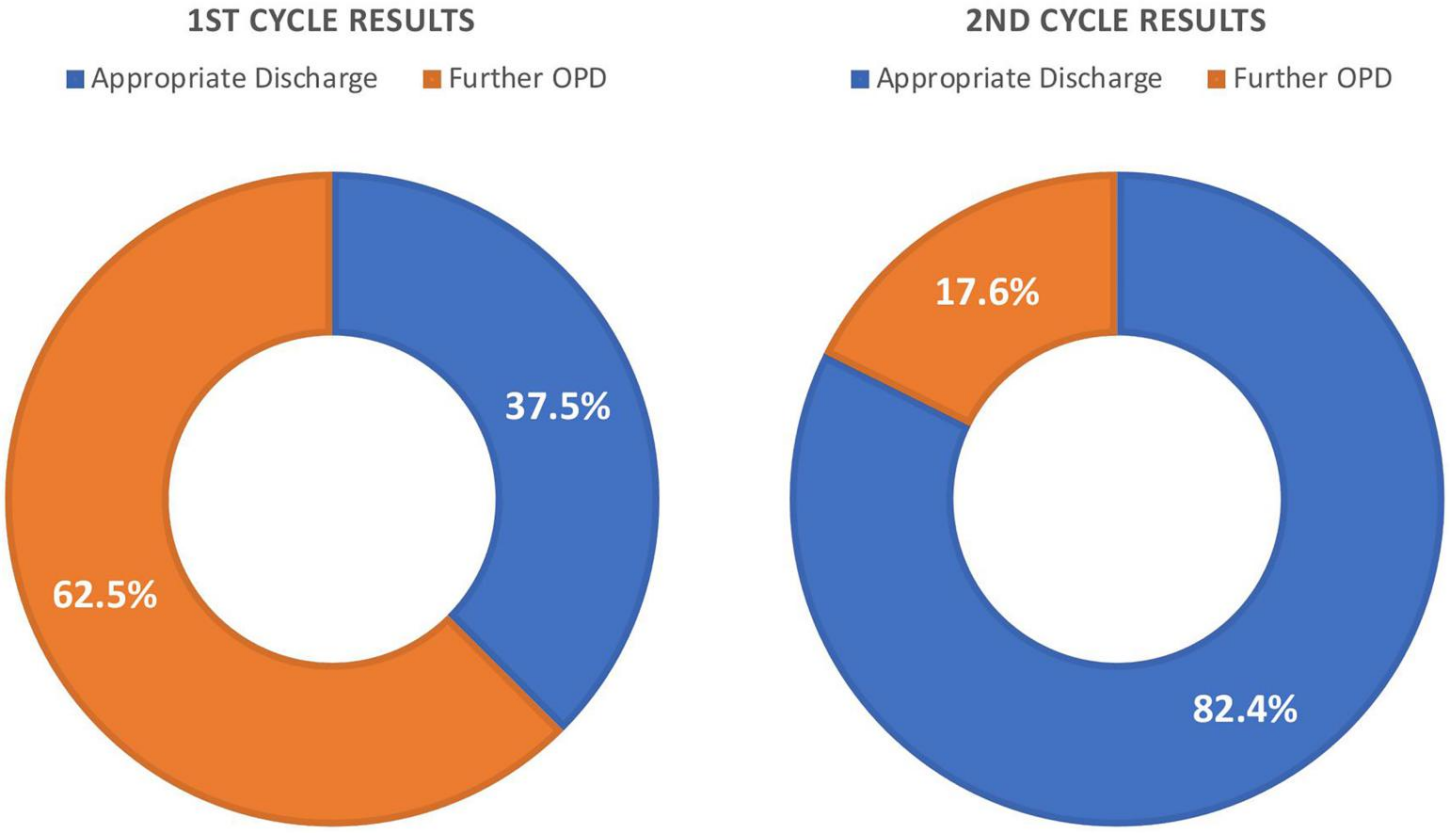
Illustrating difference between first and second cycle results following intervention.

For our audit intervention, we allocated one of our departmental teaching sessions for junior doctors and nurse specialists to discuss the updated BAD guidelines and the various tumour factors, patient factors and margin status that are used to pathologically stage cSCC. Our staff members were also educated on the correct follow‐up timeframe for each for the pathological stages. A poster of the BAD guidelines illustrating follow‐up plans for each cSCC stage was distributed amongst the NCHDs.

During our second audit cycle, we collected data from January to March 2023 again looking at new cSCC diagnosed in our department. There were 17 low‐risk cSCC patients out of a total of 55 cSCCs identified over this 3‐month period. 3 (17.6%) of patients were offered unnecessary OPD follow up while 14 (82.4%) of patients were appropriately discharged following their single post‐treatment appointment (Figure 1).

Unnecessary clinical follow up places a burden on our busy OPDs. It also contributes to patient anxiety surrounding cancer follow up^6^ and can also be an inconvenience if they need to organise transport or take time off work to attend OPD.

Educating our staff members regarding appropriate cSCC follow‐up was shown to reduce the burden on OPD and allows us to discharge our patients safely. Many studies have shown the importance of audit and teaching in reinforcing good clinical practice^7^, ^8^ while continuous staff education is imperative for reinforcing adherence to guidelines.^9^

Smak Gregoor et al postulate that overly frequent follow‐up appointments could be seen as an inefficient use of already stretched hospital budgets.^1^ Moreover, a recent systematic review by Mirali et al looked at 14 international guidelines for KC follow‐up. This study showed that there are widespread variations in KC follow‐up recommendations with no high‐quality data pointing to an optimum follow‐up schedule.^10^

The total cost of running the Plastic Surgery OPD in St Vincent's University Hospital in 2018 was €10,000,000. When we extrapolated the costs of staffing each OPD with consultants, junior doctors, nursing and administration staff and consumables, we discovered that each OPD appointment slot cost €62.94. Therefore, by reducing the number of unnecessary OPD appointments we can also reduce departmental costs going forward.

## CONFLICT OF INTEREST STATEMENT

None to declare.

## AUTHOR CONTRIBUTIONS

**Daire Goodman:** Conceptualisation (lead); Formal analysis (lead); Investigation (lead); Methodology (lead); Project administration (lead); Writing – original draft (lead); Writing – review & editing (lead). **Gary Fenn:** Data curation (supporting). **Brian Pierce:** Data curation (supporting); Resources (supporting). **Rhona Thuillier:** Data curation (supporting); Resources (supporting). **Hannah Glavin:** Resources (supporting). **Roisin Dolan:** Investigation (supporting); Supervision (supporting); Writing – review & editing (supporting).

## ETHICS STATEMENT

Not applicable.

## Data Availability

The data underlying this article will be shared on reasonable request to the corresponding author.
